# Supplementary material for: The influence of personal care products on ozone-skin surface chemistry
Source: PLoS One. 2022 Sep 29;17(9):e0268263. doi: 10.1371/journal.pone.0268263 (PMC9522313; doi:10.1371/journal.pone.0268263)
Supplement: S1 File — (DOCX) [file pone.0268263.s001.docx]

**Supplementary Information for**

**Yields and variability of ozone reaction products from human skin**

Glenn Morrison^1*^, Azin Eftekhari^1^, Aixing Fan^2^, Francesca Majluf^3^, Jordan E. Krechmer^3^

^1^ Department of Environmental Sciences and Engineering, Gillings School of Global Public Health, The University of North Carolina at Chapel Hill, North Carolina, USA

^2^ Colgate-Palmolive Co. Piscataway, NJ, USA

^3^ Aerodyne Research Inc., Billerica, Massachusetts, USA

*^*^Corresponding email: glenn.morrison@unc.edu*

Contents

[S1 Study participant recruitment and questionnaire documents 2](#_Toc89847932)

[Recruiting flyer (similar to other recruitment documents and scripts) 2](#_Toc89847933)

[Pre-enrollment Informational Interview and Questionnaire 3](#_Toc89847934)

[Pre-test interview information and questionnaire 3](#_Toc89847935)

[S2 Experimental system 4](#_Toc89847936)

[S3 Gases and Chemical Analysis 7](#_Toc89847937)

[S4 PTR-TOF-MS information including calibration mixture and sniff tests 9](#_Toc89847938)

[S5 Mass of applied lotion 11](#_Toc89847939)

[S6 Experimental sequence and system diagrams 11](#_Toc89847940)

[Example experimental procedure for one participant (with approximate timing). 11](#_Toc89847941)

[S7 Dilution of skin lipids and deposition velocity 13](#_Toc89847942)

[S8 Reaction products of octinoxate ozonation 15](#_Toc89847943)

[S9 Time constant for 6MHO approach to steady-state 17](#_Toc89847944)

[S10 Thickness of SSL and estimate of change in partition coefficient 21](#_Toc89847945)

# S1 Study participant recruitment and questionnaire documents

### Recruiting flyer (similar to other recruitment documents and scripts)

**Seeking participants for research study**

Researchers in the Environmental Sciences and Engineering Department are seeking individuals to participate in a research study of whether certain lotions protect skin from air pollution. In this study, a small area, about the size of a quarter, of skin of the forearm is coated with lotion. Air containing ozone, a pollutant, is passed over the skin area and the air is tested. We are looking for certain chemical products generated when ozone reacts with skin oil. On the day of the study, you would shower as usual, but refrain from using personal care products until after the study, which would take place later in the day. You would spend about 2 hours in the study itself and be given a $50 gift card for your participation. The study will take place in the Michael-Hooker research labs during the week of July 8, 2019. You must be 18 years or older and have normal skin. If you are interested in participating, please contact:

**Glenn Morrison, Professor**

**Environmental Sciences and Engineering**

**Gillings School of Global Public Health**

**University of North Carolina at Chapel Hill**

**(919) 966-5452**

[**glenn.morrison@unc.edu**](mailto:glenn.morrison@unc.edu)

Institutional Research Board

IRB # 18-3126

IRB Contact: CB 7097

720 Martin Luther King Jr. Blvd.

Bldg # 385, Second Floor

Chapel Hill, NC 27599-7097

Ph: 919-966-3113

Fax: 919-966-7879

### Pre-enrollment Informational Interview and Questionnaire

The purpose of this study is to determine whether skin lotions can reduce the amount of oxidation on skin due to exposure to air pollution. Participants will wear a small disk on their arm (like a wrist watch). Air with ozone will flow through his disk, over skin, and out to a device that measures chemicals that indicate whether ozone has reacted with skin oils. Six different skin locations will be tested, some of which will be coated with lotions. The lotions have different formulations, some intended to prevent ozone from reacting with skin.

You have indicated that you are interested in participating in this study. Before we can enroll you in the study, we need to make sure that you meet the criteria for an acceptable participant.

1. Are you 18 years of age or over? Y/N
2. Do you have a skin condition such as eczema or dermatitis? Y/N
3. Do you have skin sensitivities to lotions or other personal care products? Y/N
4. Will you be available to participate in this study between April 22 and May 10? Y/N

Prior to enrollment, a participant was required to complete and sign and informed consent form.

On the day of the experiment, the participant completed a brief questionnaire (see below) to verify that they have followed the preparatory procedure. Any skin reaction to the lotion or significant discomfort would halt the experiment, although this did not occur with any participants. If the participant did not comply with the pre-experimental requirements regarding showering and use of personal care products, they will be asked to return on another day or withdraw from the study; this occurred once with one subject who returned on another day. A subject that could not schedule an appointment during the testing period (a 2 week period) was withdrawn from the study; four were withdrawn for this reason.

### Pre-test interview information and questionnaire

The purpose of this study is to determine whether skin lotions can reduce the amount of oxidation on skin due to exposure to air pollution. Participants will wear a small disk on their arm (like a wrist watch). Air with ozone will flow through his disk, over skin, and out to a device that measures chemicals that indicate whether ozone has reacted with skin oils. Six different skin locations will be tested, some of which will be coated with lotions. The lotions have different formulations, some intended to prevent ozone from reacting with skin.

1. What is your age?
2. What is your sex?
3. Did you shower last night but not today?
4. What kind of soap did you use to wash your skin?
5. Have you applied any personal care products after showering and if so which kinds?
6. Do you have any concerns or questions regarding the test or the consent form?

# S2 Experimental system

Ozone exposure system. Shown in Figure S1a is how a sampler is attached to the inner forearm when a test it taking place. Shown in Figure S1b is the overall experimental system with valves positioned to expose the skin to ozone and Figure S2 shows the cross-sectional detail of the surface samplerUltrapure air was delivered through bubblers to increase the inline humidity to approximately 60% RH. Three valves directed the air as described in the ozone exposure sequence shown in Table S1 and shown with valve positions in Figures S3-S6 The sequence is intended to: 0) determine background ozone mixing ratio in the absence of skin by flowing ozone through sampler while it is sealed to the back of another clean PFA sampler, 1) flow air through sampler, over skin, to generate an ozone-free skin-emissions sample for the proton transfer reaction time-of-flight mass spectrometer (PTR-TOF-MS), 2) stabilize ozone, generated using an ultraviolet light ozone generator (ThermoElectron Corporation Model 101), without exposing skin to ozone (bypassing sampler) and determine the upstream ozone mixing ratio; 3) expose skin to ozone, measure downstream ozone and measure volatile ozonolysis products in the PTR-TOF-MS, 4) continue flowing air through the surface sampler while measuring ozonolysis products without ozone present (decay period) and 5) bypass sample cell to provide PTR-TOF-MS with ozone-free clean air. The residence time of the flux cell and sampling system is approximately 1 second, limiting the influence of gas-phase chemistry on the product composition that reaches the detector. This ozone exposure sequence is applied to each of 6 locations on the skin; the locations and lotion application sequence are described in S6


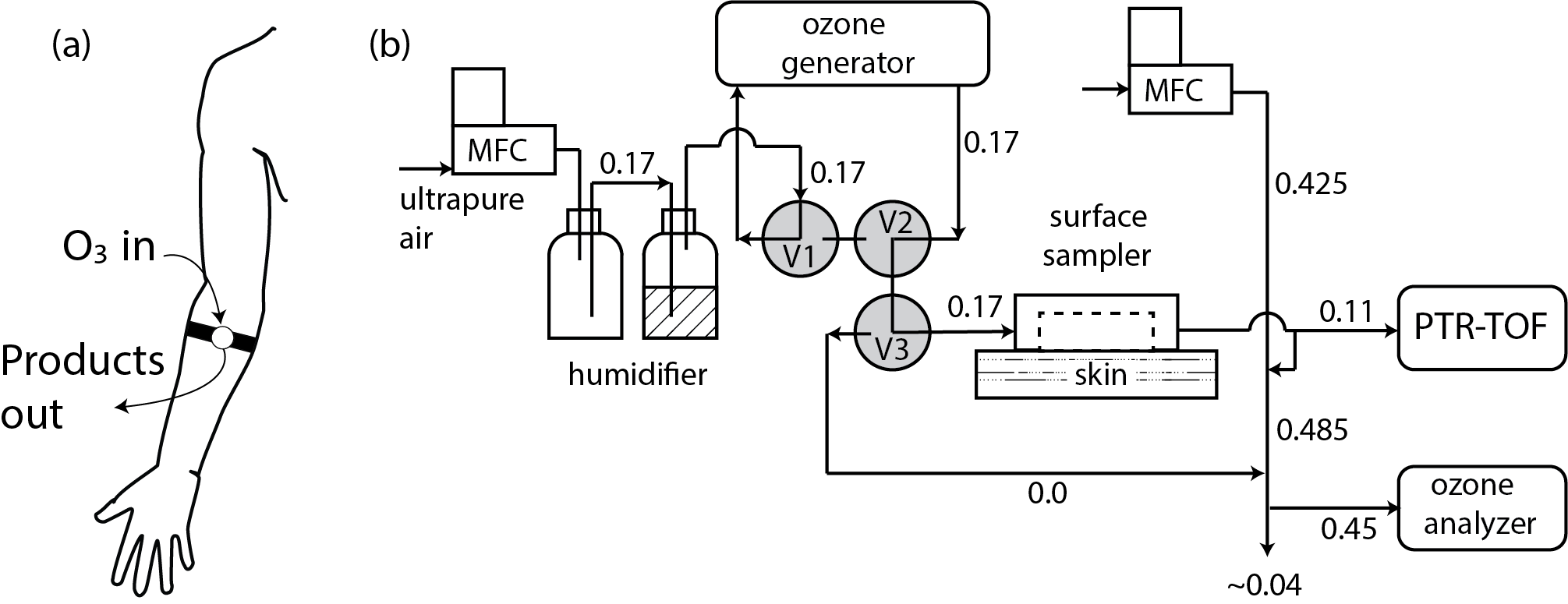


**Figure S1**. a) Sampler location shown attached to right forearm. b) System diagram showing step 3 of exposure sequence. Valves V1, V2 and V3 are shown in the on position (ozone directed to the skin). Diagrams showing other on/off states are shown in Supplementary Information Figures S1-S4. MFC= mass flow controller; PTR-TOF-MS = Vocus proton transfer reaction time-of-flight mass spectrometer.

**
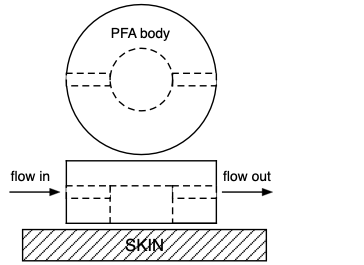
**

**Surface sampler dimensions**

- Outer diameter = 3.81 cm
- Overall height=1.59 cm
- Cavity diameter= 1.59 cm
- Cavity depth =0.95 cm
- Entrance and outlet holes both accommodate 1/8” tubing (0.318 cm diameter)

**Figure S2**. Design and dimensions of surface sampler.

**Table S1**. Ozone exposure sequence for one skin location

| Step | Time (min:sec) | (V1,V2) | (V3) | Notes |
| --- | --- | --- | --- | --- |
| 0 | -05:00 | On | On | Sampler is sealed to the back of another sampler, with ozone on. This provides a value of *C*_blanl_ |
| 1 | 00:00 | Off | On | Cell is attached to forearm and clean air without ozone flows through cell. |
| 2 | 01:00 | On | Off | Flow is directed away from the cell and ozone path is opened |
| 3 | 03:00 | On | On | Flow is directed to cell with ozone |
| 4 | 08:00 | Off | On | Ozone is off, but flow is still directed through cell |
| 5 | 10:00 | Off | Off | end sequence |

**
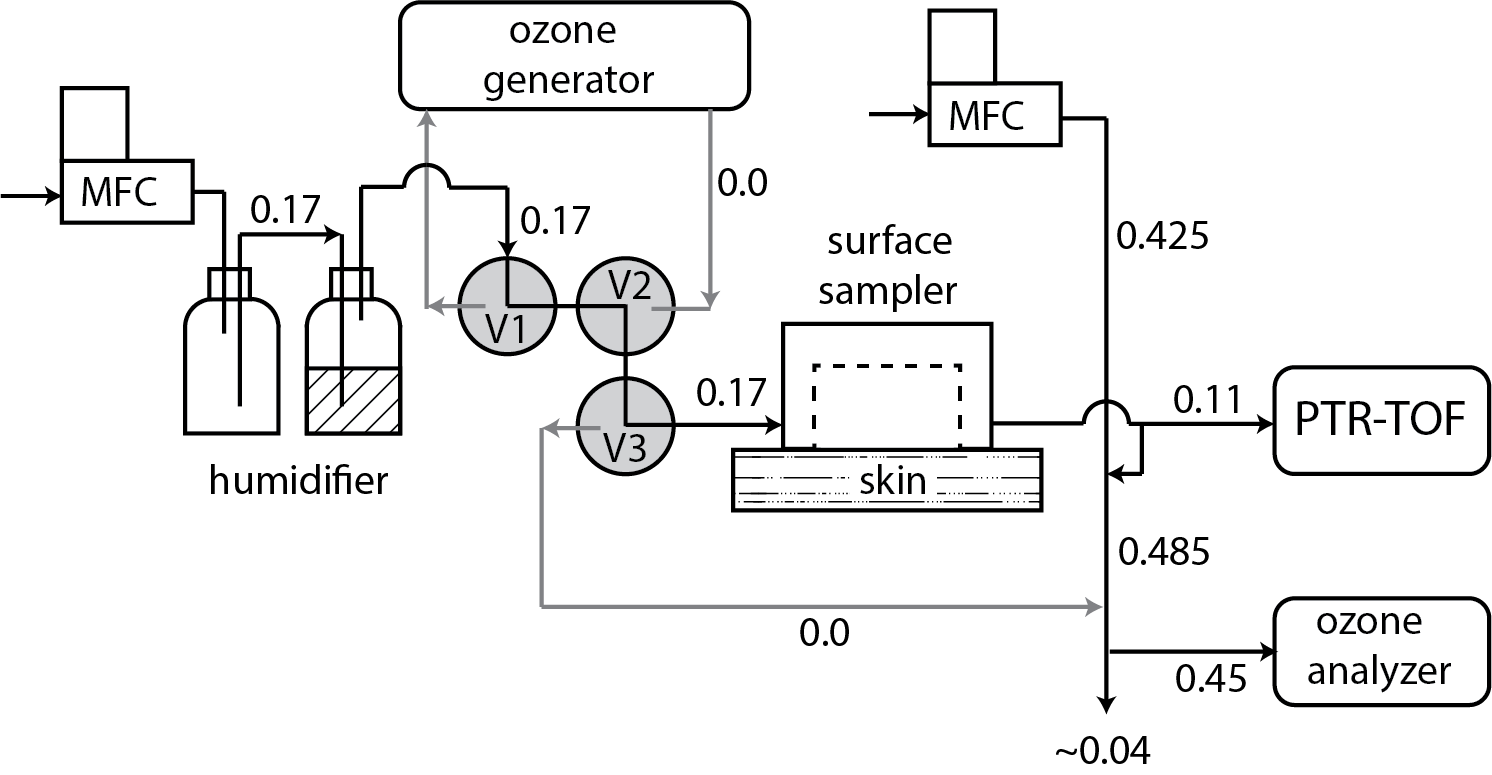
**

**Figure S3.** Step 1 system diagram. Valves V1 and V2 and are shown in the off position, while V3 is on (ozone-free air directed to the skin). This represents step 1 of the exposure sequence (time 0:00). Black lines represent paths with flow, gray lines paths without flow.


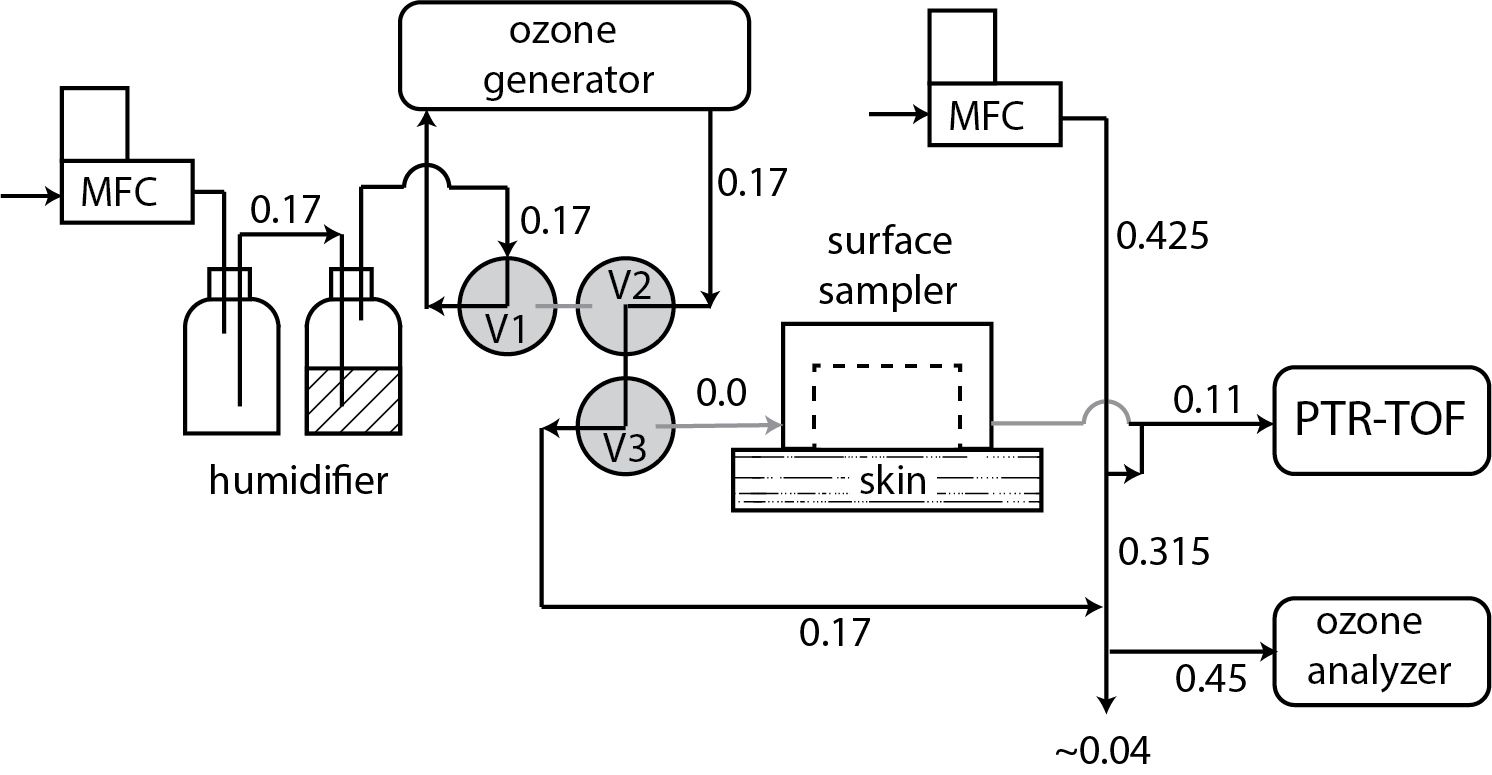


**Figure S4**. Step 2 system diagram. Valves V1 and V2 and are shown in the on position, while V3 is off (ozone bypasses skin). This represents step 2 of the exposure sequence (time 1:00). Black lines represent paths with flow, gray lines paths without flow.


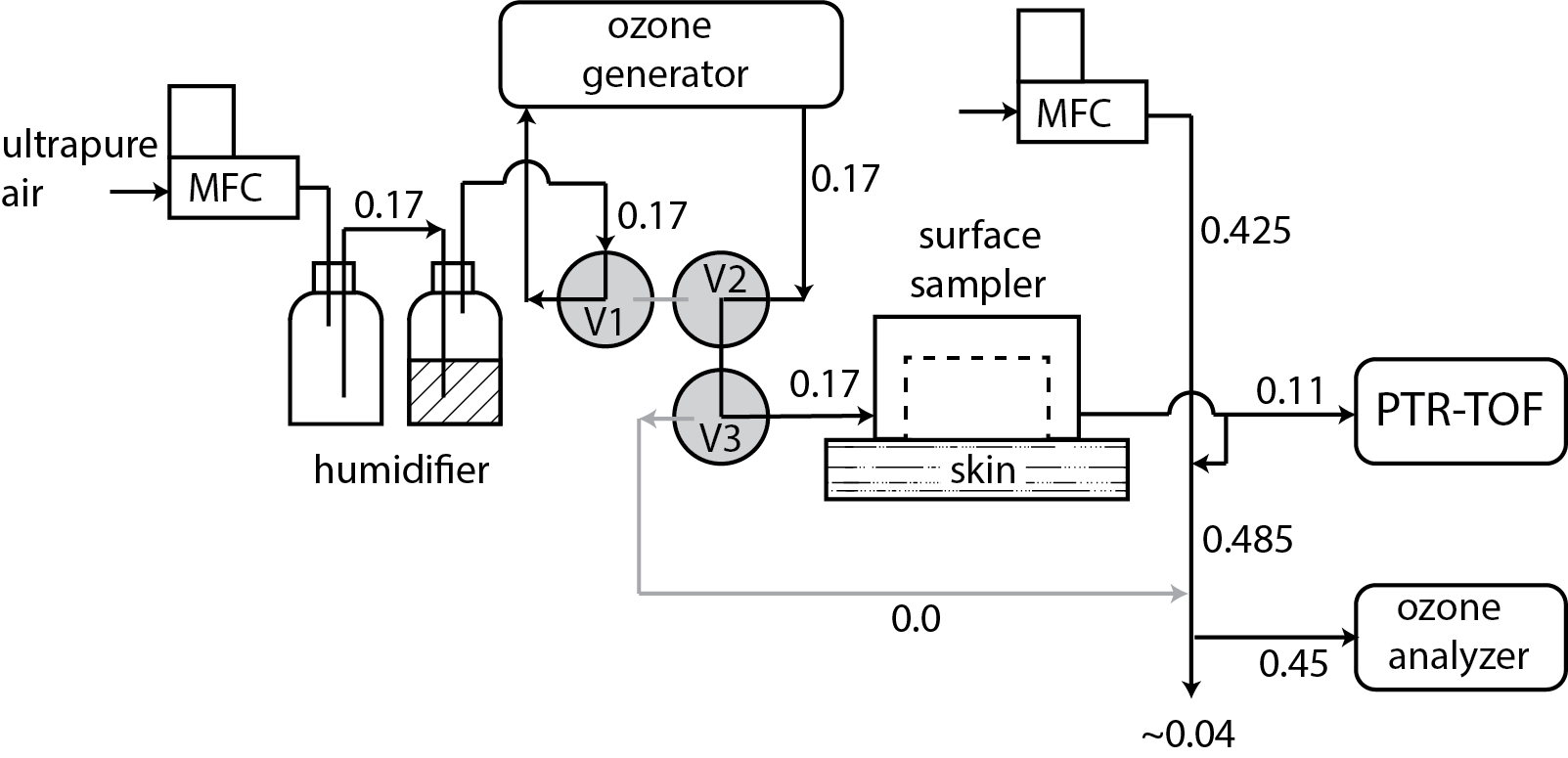


**Figure S5** Step 3 system diagram. Valves V1 and V2 and are shown in the on position, and V3 is on (ozone directed to the skin). This represents step 3 of the exposure sequence (time 3:00). Black lines represent paths with flow, gray lines paths without flow.


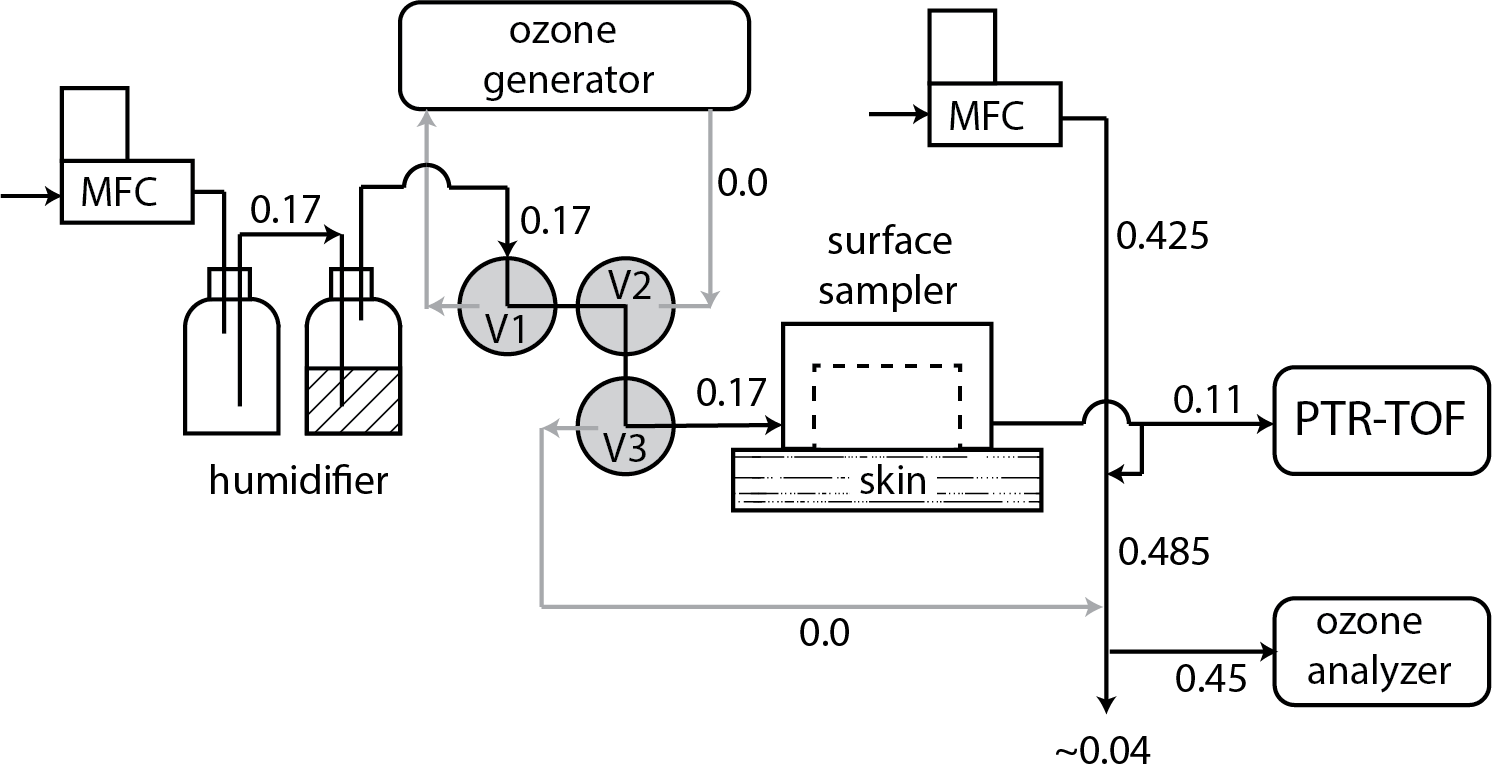


**Figure S6**. Step 4 system diagram. Valves V1 and V2 and are shown in the off position, while V3 is on (ozone-free air directed to the skin). This represents step 4 of the exposure sequence (time 8:00) which is the same as Step 1. Black lines represent paths with flow, gray lines paths without flow.

# S3 Gases and Chemical Analysis

The principal measurements are mixing ratios of ozone and gas-phase compounds that are generated by ozone reactions with skin lipids. The ozone mixing ratio (ppb) was measured using a UV-photometric ozone analyzer (Monitor Labs ML9811 Photometric Ozone Analyzer with analog output logged externally by DATAQ module and computer; limit of detection 1 ppb, uncertainty under experimental conditions with dilution ~4 ppb), both upstream and downstream of the skin flow-cell. The typical ozone concentration over the skin was 100-120 ppb. To reduce the potential for leaks, we limited the flow of gas through the surface sampler to 0.17 L/min. As shown on Figure 1b, a large fraction of this was required by the PTR-TOF-MS (0.11 L/min). The remaining flow (0.06 L/min) was delivered to the ozone analyzer but had to be diluted with 0.425 L/min of clean air to meet the flow requirement of the analyzer. The final dilution ratio was 8.1:1 and this ratio was applied to the measured mixing ratio to calculate the actual inlet or outlet ozone mixing ratio.

The gaseous products of ozone chemistry were quantified at the surface sampler outlet using a Tofwerk Vocus proton-transfer reaction time-of-flight mass spectrometer (Vocus PTR-TOF-MS) which uses protonated water that reacts with compounds of interest. The PTR-TOF-MS calibration procedure is detailed in the Supplementary Information section S3. Some primary, secondary and tertiary products of ozone chemistry with squalene and triglycerides are shown in Figure 2a and 2b respectively; because the residence time of the system is very short, primary reaction products with squalene, condensed oxidized squalene and other surface reactants such as triglycerides are anticipated to be the most prominent products. Results were converted to mixing ratio (ppb) for products including 6-methyl 5-hepten-2-one (6 -MHO), geranyl acetone (GA) and decanal. We were unable to adequately quantify acetone due to high background levels. Mixing ratios of two compounds, GA and 6-MHO, were corrected based on observed fragmentation ratios during pure-compound “sniff” tests (see Supplementary Information Section S3) and further corrected based on observed uptake and oxidation taking place in the sample line (see Results). The mixing ratios were then normalized by the inlet/outlet difference in the ozone mixing ratio (ppb; limit of quantification of difference ~2 ppb, uncertainty in difference ~5%) to generate a surface-specific yield. Note that the calibration factors for these compounds have a higher uncertainty since a calibration standard with the majority of oxidation products was not available at the time of the experiment.

# S4 PTR-TOF-MS information including calibration mixture and sniff tests

The design and function of the Vocus Proton-Transfer-Reaction Time-of-Flight Mass Spectrometer (Vocus PTR-TOF-MS) is described in more detail in Krechmer et al.(Krechmer et al. 2018) In this work, the focusing ion molecule reactor (fIMR) was operated at a pressure of 1.5 mbar, an axial voltage difference of 475 V, and a drift tube temperature of 100 C, corresponding to an E/N of ~160. While this will result in more fragmentation than a more typical and lower E/N value, we do not expect this to affect the conclusions of this work. Further, using standard injections, we measured the fragmentation pattern for critical compounds of interest including GA and 6-MHO and corrected the calibration factors accordingly.

Data was recorded and saved at a 1 Hz time resolution and analyzed using the Tofware software (Aerodyne Research Inc. and TOFWERK) within Igor Pro (WaveMetrics).

The Vocus PTR-TOF-MS was calibrated every two hours with a 16-component VOC standard mixture containing around 1 ppm of each compound. The 16-components consisted of common outdoor atmospheric volatile constituents of varying functional groups (e.g. acetonitrile, acetone, benzene, isoprene, methyl ethyl ketone, etc.). During each calibration period, the inlet was overflowed with the VOC mixture diluted 100x into zero air, resulting in sampling approximately 10 ppb of each VOC standard. The zero air used for dilution and background measurements was generated by pushing room air through a hydrocarbon scrubber (Vici Metronics). The Vocus PTR-TOF-MS has no humidity dependence and thus no RH-dependent calibrations were performed. Background measurements were performed before and after every calibration period.

The sensitivity of each sampled analyte molecule is a linear function of its proton capture rate coefficient with the hydronium ion (Sekimoto et al. 2017; Yuan et al. 2017) The response factors of compounds at a 16.4 KHz TOF extraction frequency in the standard mixture were plotted against their known $k_{cap}$and fit to a linear regression (figure S5). Good agreement (r^2^ = 0.92) was obtained between the measured response factors and those using their known $k_{cap}$.


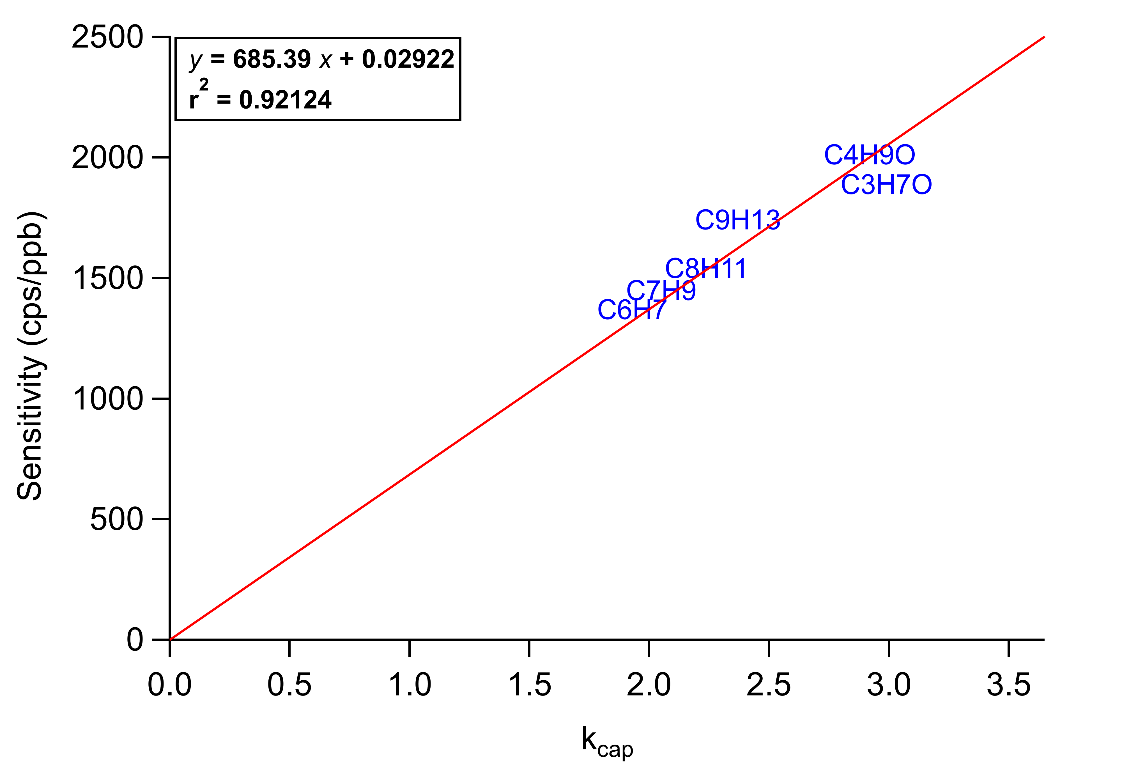


**Figure S7:** Response factor of calibration compounds versus their proton-capture rate coefficient ($k_{cap}$). Slope of 685.39 was used to obtain the response factor of other VOCs by multiplying it by their estimated $k_{cap}$. Calibration compounds affected by significant uncorrected fragmentation or mass-to-charge-dependent transmission through the quadrupole ion guides were excluded from this analysis.

The $k_{cap}$of compounds not found in the standard mixture can be estimated using the polarizability and dipole moment of the elemental formula extracted from the mass spectrum using a previously developed parameterization(Sekimoto et al. 2017), which is then adjusted by the following equation, where $f_{VOC}$is the estimated response factor of a given VOC in counts-per-second per ppb, $m$ is the slope on figure S5, and $k_{cap, VOC}$is the estimated $k_{cap}$for the given VOC.

$f_{VOC}=m\times k_{cap, VOC}$ (S1)

To correct for the fragmentation of 6MHO and GA, non-quantitative amounts of chemical standard were introduced to the instrument. Each standard introduction consisted of the sampling the headspace of the pure chemical in question by quickly opening and closing the bottle in front of the Vocus PTR-TOF-MS’s inlet. The procedure was repeated three times and the average ratio between the parent molecule and its fragment was calculated. Table S2 below lists the resulting fragment-to-parent ratios for both 6-MHO and GA for the PTR-TOF-MS source settings used in this experiment.

Table S1. Ratio of fragment-to-parent signal in counts-per-second.

| Compound | Parent Ion | Fragment Ion | Fragment-to-parent signal ratio |
| --- | --- | --- | --- |
| 6-MHO | C_8_H_14_OH^+^ | C_8_H_12_H^+^ | 4.21 |
| GA | C_13_H_22_OH^+^ | C_13_H_20_H^+^ | 3.55 |

Due to the unusually high E/N used in this study, fragmentation for 6-MHO and GA were higher than observed in other studies (e.g. Wisthaler and Weschler, 2010(Wisthaler and Weschler 2010)). The fragmentation ratio is dependent on the electric field to number density ratio, or *E/N*, typically represented in units of Townsend (Td). Figure S4. shows a plot of the relative amount of fragmentation for geranyl acetone as a function of PTR-TOF-MS E/N. To take these data, the PTR-TOF-MS axial voltage and temperature were held constant, and the source pressure was varied to keep transmission constant while changing E/N.


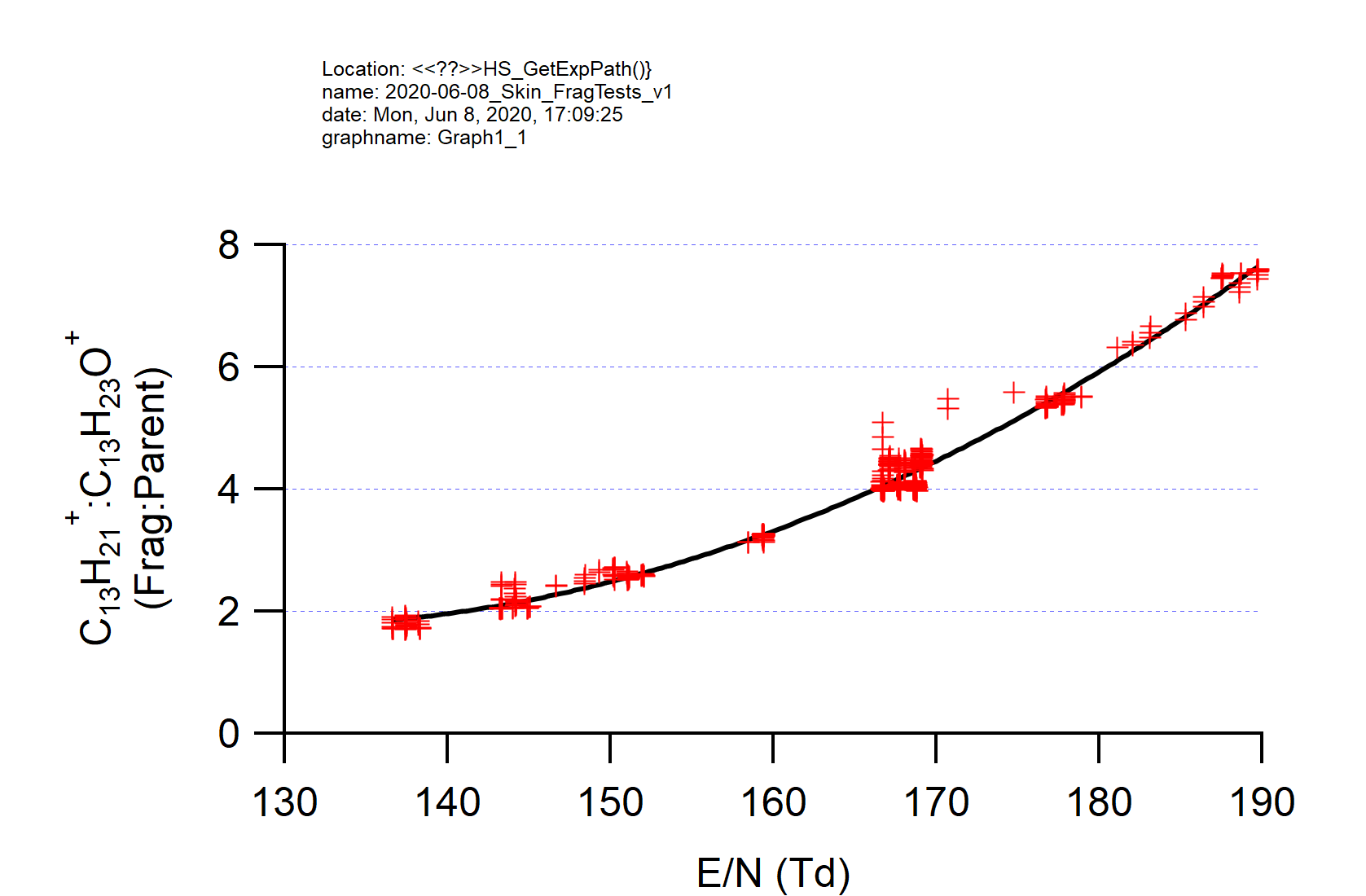


**Figure S8**. Fragmentation for geranyl acetone as a function of PTR-TOF-MS source E/N, represented by the ratio of the fragment to the parent ion.

# S5 Mass of applied lotion

**Figure S9**. Average mass applied for lotions 1-5. Error bars represent 1 standard deviation.

# S6 Experimental sequence and system diagrams

## Example experimental procedure for one participant (with approximate timing).

9:00 Pre-test interview information and questionnaire.

9:10 Participant is shown to the lab and sits in chair provided.

9:10 Lotion 3 (lotions pre-randomized for each participant) is applied to forearm location L2. Note, precise forearm locations were determined based on individual characteristics of the participant (comfort, arm hair, etc.), but were generally consistent across participants.

9:20 Location L1 (uncoated control) is covered by a flow cell which is held down gently with elastic gauze. Clean, humidified air without ozone is allowed to flow through cell. Instruments (VOCUS, ozone analyzer) are sampling from the outlet air continuously. See *Ozone exposure sequence.* Table S.2 below for specifics.

9:20 Lotion 4 is applied to forearm location L3.

9:30 Cell is moved from Location L1 to L2. At this time, Lotion 3 has had 20 minutes to dry. *Ozone exposure sequence* (below) is initiated.

9:30 Lotion 1 is applied to forearm location R4.

9:40 Cell is moved from Location L2 to L3. *Ozone exposure sequence* is initiated.

9:40 Lotion 5 is applied to forearm location R5.

9:50 Cell is moved from Location L3 to R4. *Ozone exposure sequence* is initiated.

9:50 Lotion 2 is applied to forearm location R6.

10:00 Cell is moved from Location R4 to R5. *Ozone exposure sequence* is initiated.

10:10 Cell is moved from Location R5 to R6. *Ozone exposure sequence* is initiated.

10:20 Cell is removed from arm and participant may leave.

# S7 Dilution of skin lipids and deposition velocity

We observed that lotions reduced the ozone-to-skin deposition velocity by 12 to 25% and consider whether this makes sense with respect to the anticipated dilution of skin lipids with relatively inert lotion ingredients. The natural thickness of skin lipids is approximately 1 µm (Weschler and Nazaroff 2012). When a lotion is applied to the skin, the total thickness of the skin surface layer will increase somewhat depending on how much of the lotion ingredients remain on the surface after “drying”. For Lotion 1 of this experiment, we assume that the non-volatile fraction comprises about 20% based on experience with other lotions tested in our lab (Eftekhari and Morrison, 2021); however, we did not measure this fraction directly. We also assume that the non-volatile fraction has the same density as the first non-water ingredient, propylene glycol (1.04 g/m^3^). Therefore, application of 2mg/cm^2^ is equivalent to increasing the skin surface layer thickness to about 5 µm and thereby diluting the skin surface lipids by a factor of 5.

If the skin lipids are being diluted by a factor of 5, why is ozone flux to skin only reduced by, at most, 25%? This is most likely due to the fact that the system is transport limited, i.e. gas-to-skin transport of ozone represents the primary resistance to over uptake of ozone. The effective deposition velocity is defined as described in the main text equation (2) with mean results ranging from a low of 8 m/h (Lotion 3) to 12 m/h for bare skin.

Cano-Ruiz et al. (1993) derived a general equation for the deposition velocity of a reactant to a flat surface. This combines the transport limited deposition velocity (*v_t_*; independent of surface reactivity, but dependent on gas-side fluid mechanical conditions), and the ozone-interface reaction probability ( *γ*; independent of external mass transfer conditions/limitations). This reaction probability is the probability that the reactant (ozone in this case) will react (instead of eventually returning to the gas phase), after it has collided with the surface. The deposition velocity is then given by (Cano-Ruiz-et al. 1993):

$v_{d}=\left[ \frac{1}{v_{t}}+\frac{4}{\left\langle v \right\rangle\gamma} \right]^{-1}$ (S3)

This can be visually represented as in Figure S10. The bare-skin average value of *v_d_* in our study was about 12 m/h; assuming for the moment that this is the actual transport limited deposition velocity (*v_t_*), then the deposition velocity for the cell as a function of the surface reaction probability is shown as the blue line. For a log­_10­_(γ) between -3.5 and 0, the deposition velocity is insensitive to the surface reaction probability. For lower values of γ, the deposition velocity becomes more sensitive until log­_10­_(γ) < -5; at this point, the deposition velocity is directly proportional to γ. Also shown on the figure are three scenarios in which the reaction probability is reduced by a factor of 5 (corresponding to anticipated dilution by inert lotion components).

- If the initial log­_10­_(γ) = -3, then a reduction by a factor of 5 (log­_10­_(γ) = -3.7) results in a reduction in the deposition velocity of only 12%.
- If the initial log­_10­_(γ) = -4, then the reduction in deposition velocity is about 52%. This demonstrates how sensitive the system is to initial conditions.
- If the initial log­_10­_(γ) = -5, then the reduction in deposition velocity is about 76%. This is nearly the factor of 5 reduction that would be expected if the diluted system is limited by the rate of reaction of ozone with skin lipids.


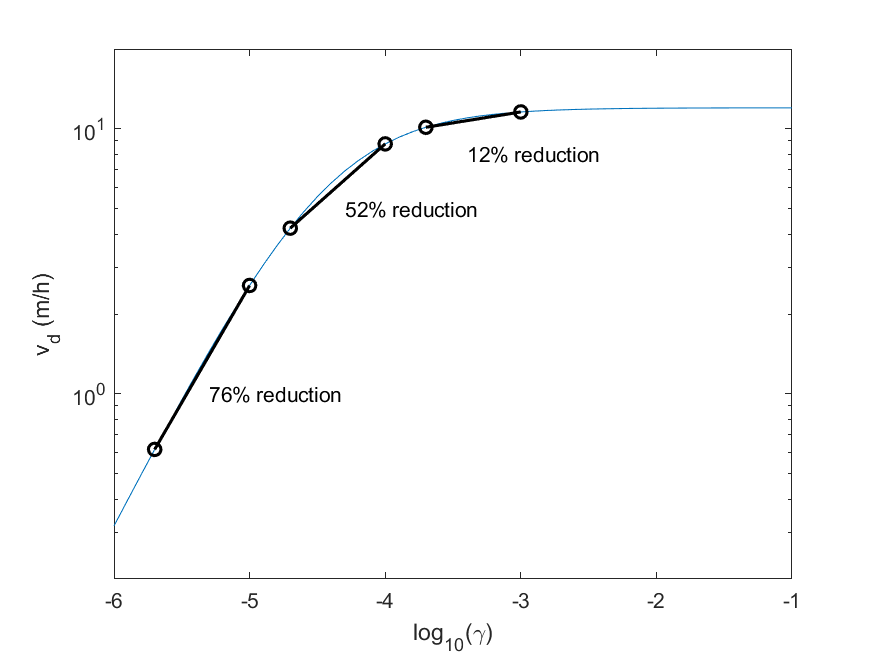


**Figure S10**. Deposition velocity as a function of log10 (γ), for a transport-limited deposition velocity, *v_t_*, of 12 m/h.

The reaction probability of squalene is approximately 5 × 10^-4^ (Wells et al. 2008) and is likely the most reactive species present in skin lipids. At first glance, the skin lipid matrix might be expected to have a slightly lower reaction probability if only considering reactions with a flat surface since other lipids present are less reactive. In other words, averaged over all lipids present, the reaction probability would be < 10^-4^. However, this does not account for the fact that ozone can diffuse below the first layer of lipid molecules and react with those below. This increases the availability of reactive lipids and increases the “effective” ozone reaction probability of surface(Moise and Rudich 2002; Knopf et al. 2005). The same is true when the lipids are diluted with a lotion. It is therefore plausible for a skin surface layer that is diluted with inert lotion ingredients can still have a fairly high effective reaction probability. Furthermore, some of the lotion components may NOT be inert, which would add to the reactivity of the lotion-lipid mixture. This appears to be the case for Lotion 4 with antioxidant additives.

# S8 Reaction products of octinoxate ozonation


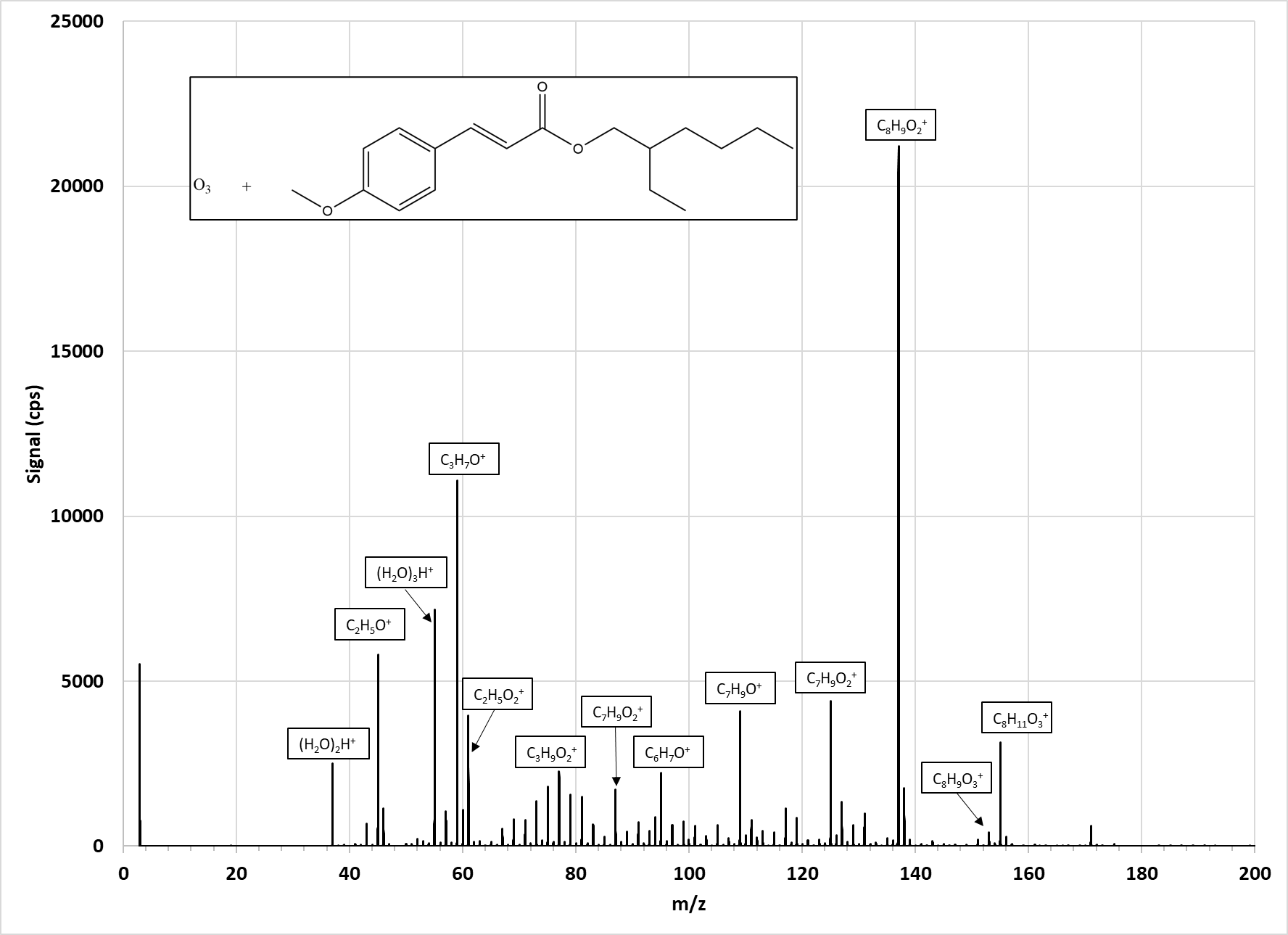


**Figure S11**. PTR-TOF-MS spectra of air (with ozone) after passing over octinoxate; background signal associated with emissions in the absence of ozone have been subtracted from the spectra.


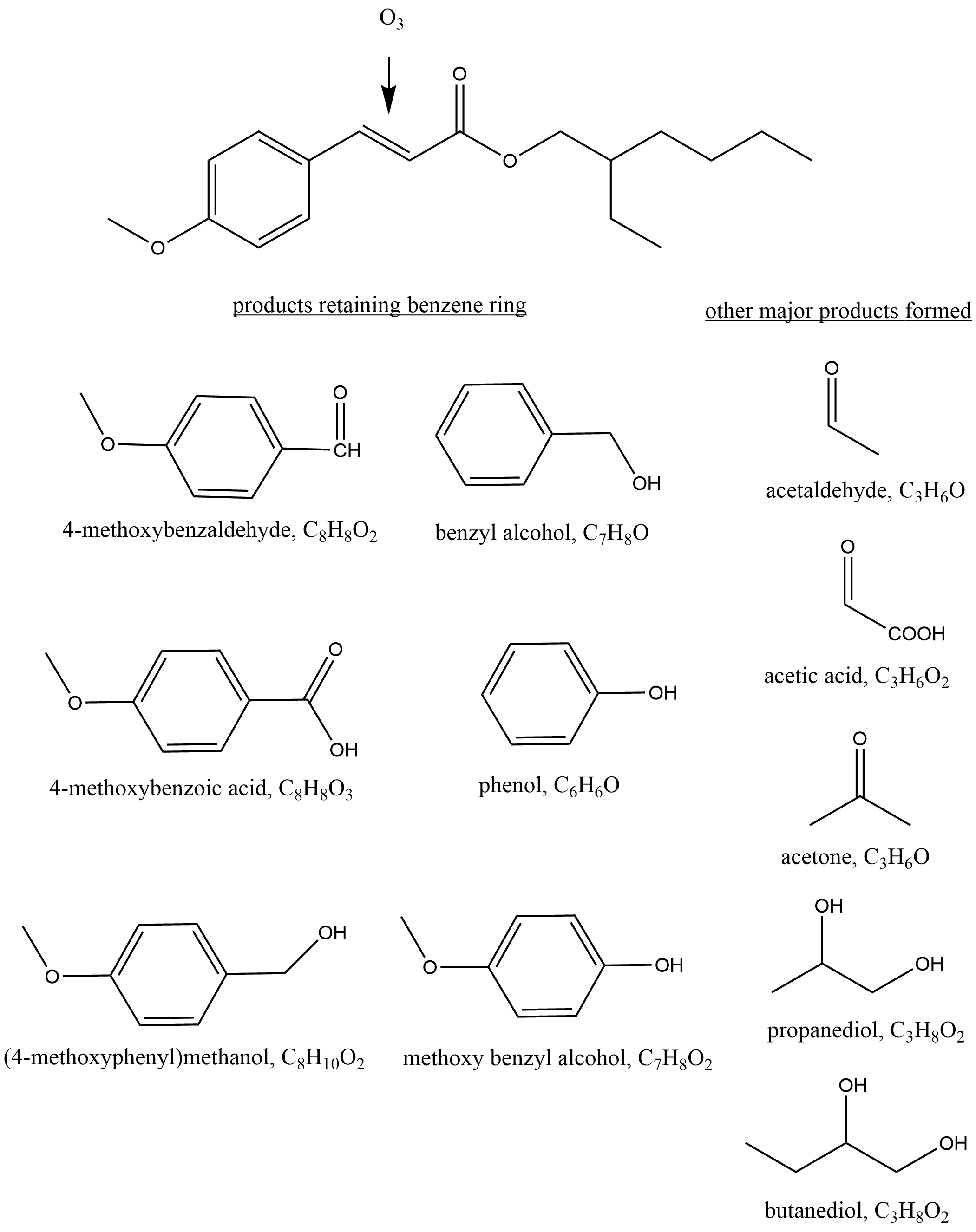


**Figure S12**. Products of the reaction of ozone with octinoxate

# S9 Time constant for 6MHO approach to steady-state

During the 5-min ozone-on period, the concentration of 6MHO leaving the surface sampler rises to approach steady state. For bare skin and lotion 5, this approach is slower than for lotions 1-4. The characteristic time to rise can be determined in multiple ways (as described here), but the physical mechanism controlling this dynamic is likely the result of multiple sorptive sinks. Here, we discuss only two: delayed desorption from the skin surface film (SSF; composed of skin surface lipids, SSL, and low-volatility lotion components) and partitioning delay associated with the sampling system itself.

The emission rate of 6MHO from the skin surface film will be influenced by the rate of formation by reaction of squalene with ozone, the equilibrium partitioning between the skin surface film and air (as parameterized by a partition coefficient) and diffusive flux of reactants and products through the skin surface layer and possibly through the stratum corneum. To simplify this system as much as possible we assume that the formation rate of 6MHO is constant and uniformly distributed through the SSF, and diffusion within the SSF is rapid, surface-to-gas mass transport is the primary mass transfer resistance to emissions. A mass balance on the skin surface layer is then given by,

$A_{skin}d_{SSF}\frac{dC_{SSF}}{dt}=R-\frac{v_{t}C_{SSF}A_{skin}}{K_{SSF-g}}$ (S4)

Where,

*A_skin_* is the cross-sectional area of skin, m^2^

*d_SSF_* is the depth of the skin-surface film, m

*C_SSF_* is the concentration of a reaction product (6MHO in this case) in the skin surface layer, µg/m^3^

*R* is the formation rate of the product, µg/m^3^/h

*v_t_* is the deposition velocity or gas-side mass-transfer coefficient, m/h

*K_SSF_* is the partition coefficient between the SSF and the overlying gas, (µg/m^3^)_SSL_/(µg/m^3^)_gas_ and defined as the ratio of concentrations in the SSF and gas at equilibrium:

$K_{SSF-g}=\left[ \frac{C_{SSF}}{C_{gas}} \right]_{equilibrium}$ (S5)

Equation S.X can be solved for *C_SSF_* as a function of time. Assuming *C_SSF_* = 0 at t=0 s,

$C_{SSF}=\frac{R*K_{SSF-g}}{v_{t}A_{skin}}\left( 1-e^{-t\frac{v_{t}}{d_{SSF}K_{SSF-g}}} \right)$ (S6)

The characteristic time for the SSL to approach steady state is then (from the exponential expression),

$\tau=\left( \frac{v_{t}}{d_{SSF}K_{SSF-g}} \right)^{-1}$ (S7)

Expression S.X can be normalized by the steady-state concentration and simplified to

$\frac{C_{SSF}}{C_{SSF\_ss}}=\left( 1-e^{-t/\tau} \right)$ (S8)

This is also equal to the normalized emission rate and the normalized gas-phase concentration assuming no other interactions with sampler surfaces. This would mean that,

$\frac{C_{gas}}{C_{gas\_ss}}=\left( 1-e^{-t/\tau} \right)$ (S9)

We found that a single exponential approach to steady-state did not adequately match the observed dynamic rise in the 6MHO concentration. To account for this discrepancy we assumed that the concentration of 6MHO measured at the instrument inlet has also been influenced by sorptive delays in the sampling system itself including surfaces of the sampler, tubing and instrument inlet. This is a more complex system but for simplicity, we allow that this represents a sequential resistance that can be modeled using a double-exponential approach to steady state. An empirical model of this system is then,

$\frac{C_{gas}}{C_{gas\_ss}}=\left( 1-e^{-t/\tau1} \right)\left( 1-e^{-t/\tau2} \right)$ (S10)

Where τ1 and τ2 represent time constants associated with two different mechanisms. Show in Figure 12 are the data and line fits to equation S10 using Matlab “fit” function. Also shown is the larger of the two time-constant values, τ1.


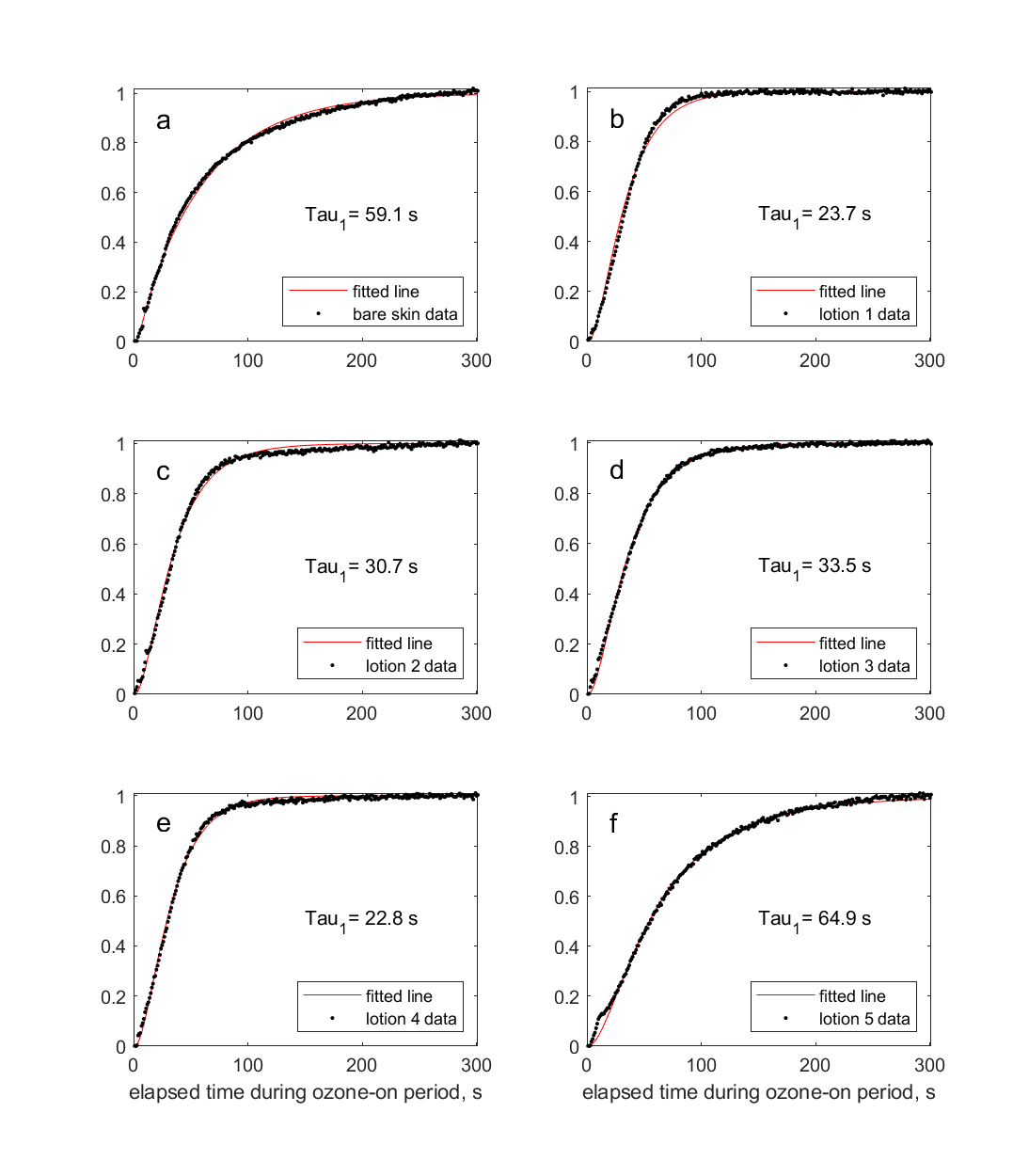


**Figure S13**. Model fit to equation S10 for 6MHO dynamic rise to steady state (average over all participants).

Shown in Figure S13 are dynamic results for geranyl acetone (GA), presented in the same manner as for 6MHO in Figure 5 of the main text. While there are, visually, differences in the results between skin and lotions 1-4, they are not as dramatic as the dynamics of 6MHO. This may be because GA sorbs strongly to all surfaces, including the sampling system. Therefore, the time delay prior to reaching steady state may be so dominated by sampling system sorption that any delays associated with skin surface sorption do not significantly influence the dynamic rise. The very small signal for GA emissions from lotion 5 (Figure S13c) make the normalized signal quite noisy, even when averaged over 20 subjects, and is therefore difficult to interpret.


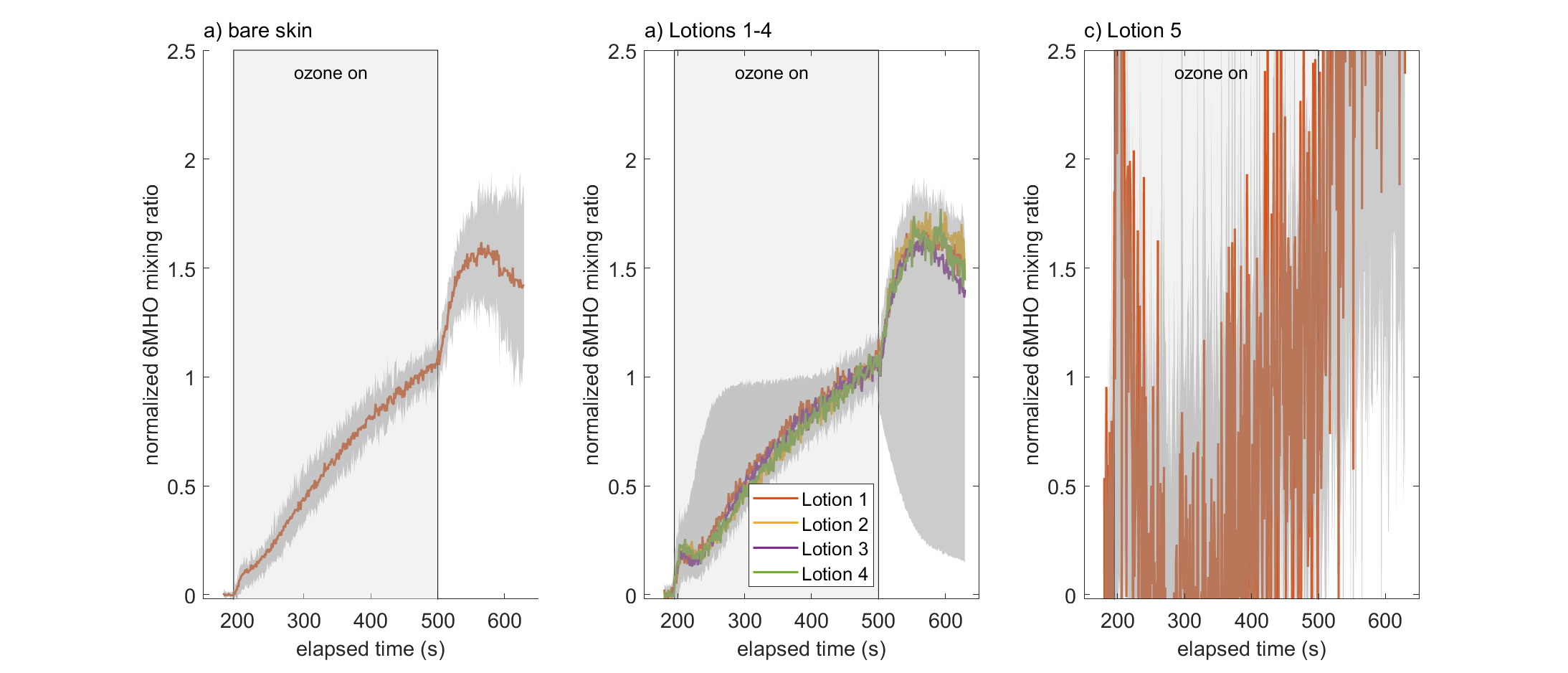


**Figure S14**. Dynamic GA signal before, during and after ozone exposure to skin, normalized by signal during the last 10 seconds of ozone exposure and averaged over all participant results. a) bare skin, b) Lotions 1-4, c) Lotion 5. Grey shaded square represents time period with ozone exposure. Darker grey shade around 6MHO curves represents 10th to 90th percentile results.

# S10 Thickness of SSL and estimate of change in partition coefficient

The natural thickness of skin lipids is approximately 1 µm (Weschler and Nazaroff 2012). When a lotion is applied to the skin, the total thickness of the skin surface layer will increase somewhat depending on how much of the lotion ingredients remain on the surface after “drying”. For Lotion 1 of this experiment, we assume that the non-volatile fraction is comprised of about 20% based on experience with other lotions tested in our lab (Eftekhari et al. 2020); however, we did not measure this fraction directly in our lab. We also assume that the non-volatile fraction has the same density as the first non-water ingredient, propylene glycol (1.04 g/m^3^). Therefore, application of 2mg/cm^2^ is equivalent to increasing the skin surface layer thickness to about 5 µm and thereby diluting the skin surface lipids by a factor of 5.

From equation S.X, we showed that the characteristic time to approach steady-state is given by,

$\tau=\frac{d_{SSF}K_{SSF-g}}{v_{t}}$ (S11)

By making some simplifying assumptions, we can estimate the lower limit on the change in the partition coefficient. First, we assume that the characteristic time shown in Figure S. X is purely associated with partitioning in the skin surface layer (this is not likely to be the case but acts as a starting point). Second, the deposition velocity is not influenced by changes in the skin surface. Therefore, by taking the ratio of equation S.X for two experiments, bare skin and lotion X, we can derive a minimum reduction in the partition coefficient due to adding lotion as,

$K_{SSF-g,lotion}=K_{SSF-g,bare\_skin}\frac{d_{SSF,bare\_skin}\tau_{lotion}}{d_{SSF,lotion}\tau_{bare\_skin}}$ (S12)

For example, $K_{SSF-g,lotion}=K_{SSF-g,bare\_skin}\left( \frac{1um*23.7s}{5um*59.1s} \right)=(0.08)K_{SSF-g,bare\_skin}$

This would mean that, by adding lotion, the surface layer partition coefficient is reduced by more than one order of magnitude. However, the first assumption is not likely valid. In reality, this time constant is certain to be associated with a combination of mechanisms. If so, then both values of the specific characteristic time, *τ*, for the skin surface layer are smaller but probably not proportionally so. Consider the situation where the delay time due to sorption/desorption from tubing is, for example, 10 s. On the assumption that the time constants are additive, subtraction of this value from each of the estimated values of *τ_lotion_* and *τ_bare_skin_* means that the ratio of these characteristic time values is even smaller and that the reduction in the partition coefficient is smaller still (*K_SSF-g,lotion_* = (0.027)*K_SSF-g,bare_skin_* for this hypothetical case).

The main conclusion is that lotion 1 (and probably in a similar way, lotions 2-4) are diluting skin lipids and reducing the 6MHO partition coefficient. This has implications for the emission rate of reaction products but also in the resulting chemical activity of oxidation products and the flux by diffusion through the stratum corneum.
